# The effect of gender on emotional reactions and perceptions when individuals meet themselves in immersive virtual reality

**DOI:** 10.1038/s41598-024-57662-2

**Published:** 2024-03-23

**Authors:** Emmanuelle P. Kleinlogel, Marianne Schmid Mast, Laetitia A. Renier

**Affiliations:** 1 CEMOI Laboratory, IAE Reunion, University of Reunion Island, 24 Avenue de la Victoire, 97400 Saint Denis, France; 2 Faculty of Business and Economics, University of Lausanne, Lausanne, Switzerland

**Keywords:** Psychology, Human behaviour

## Abstract

Immersive virtual reality-based training and research are becoming more and more popular and are in continuous development. For instance, it is now possible to be trained by one’s virtual self (i.e., doppelganger), meaning that a trainee can participate in a training program in which the trainer resembles the trainee. While past research involving doppelgangers showed promising results, findings revealed gender effects such that doppelganger-based training seems to be beneficial only for male trainees. In the present research, we contribute to this literature by investigating the emotional reactions and perceptions that people have when they meet a virtual human in immersive virtual reality. Specifically, we assess the extent to which the appearance of the virtual human (doppelganger vs. unknown avatar) and an individual’s gender influence these reactions and perceptions. We found that males felt more positive emotions toward their doppelganger than toward the unknown avatar and that females perceived their doppelganger as less competent and warm than the unknown avatar. Our findings have important practical implications in terms of virtual reality-based training and research design such that the use of a doppelganger (unknown avatar) might be recommended in a training program or research setting involving men (women).

## Introduction

Technological advances in immersive virtual reality (IVR) offer seemingly limitless research opportunities such as designing uncommon scenarios enabling out-of-the-ordinary experiences^1^. As an example, it is now possible to meet or interact with one’s virtual self (i.e., doppelganger). Most of the research involving doppelgangers is related to training. Understanding the effects of learning by exposing trainees to their virtual self is an expanding research topic and results appear promising^2–4^. However, how trainees, and more broadly speaking, how individuals react when they meet their virtual self and how they perceive their virtual self remains unclear, whereas reacting or perceiving a virtual human negatively, such as a virtual trainer in a training setting, might be detrimental for the trainee learning experience.

The aim of our research is twofold. First, we investigate individuals’ emotional reactions toward, and perceptions of, a virtual human resembling them or not (i.e., doppelganger vs. unknown avatar). We assess the extent to which individuals feel positive and negative emotions, and perceive the virtual human as competent and warm. Second, we investigate whether individuals’ gender moderates this relationship. Specifically, we focus on how individuals react toward and perceive a virtual human *at first* (doppelganger—DG vs. unknown avatar of the same gender—UA), meaning prior to measuring outcomes. For instance, it is plausible to expect that if the use of DGs induces a negative emotional reaction, this would subsequently affect individuals’ attitudes and behaviors, which would also affect the research or the training outcomes. Accordingly, the observed effect would not be due to the research topic or the content of the training per se, but due to the appearance of the virtual human that induces different reactions among individuals. It is thus crucial to investigate whether a DG (vs. UA) induces different reactions and perceptions, and whether gender matters.

The present research aims to contribute to the literature on the use of virtual reality (VR) technologies in psychological research through both a theoretical and a practical lens. On the theoretical axis, our work contributes to a better understanding of individuals’ reactions toward and perceptions of DGs in research in general as well as in research related to IVR-based training. Research in IVR often involves using virtual humans in the role of interaction partners. For instance, a virtual human might play the role of an evaluator in a self-presentation^5^ task, such as in a job interview situation. A virtual human might also adopt the physical traits of the individuals (DG) in a IVR-based training context and be used as a role model that is expected to boost trainee learning experience^4^. Nonetheless, to our knowledge research has not assessed how individuals react toward and perceive their DG, focusing only on the research outcomes.

Furthermore, research has revealed that IVR-based training involving a virtual self (i.e., one’s doppelganger) is particularly successful for male trainees^2,4^. It is plausible to argue that women react more negatively when training with their virtual self and perceive it negatively (as compared to training with an unknown avatar) due to their highly critical eye toward themselves^6–9^. From a practical perspective, such evidence would contribute to improve training and research practices. A better understanding of individuals’ IVR-based experience, through their emotions toward and perceptions of a virtual human, will lead to recommendations pertaining to research as well as to training design and its effectiveness. This also applies to the study of gender in IVR. These understandings would, on a larger scale, help to clarify the impact of individual differences in the use of IVR.

### Virtual reality as an innovative research tool

IVR technology offers almost limitless research opportunities given that it is now possible to mimic any real life situation in a virtual environment, allowing individuals to participate in a wide range of scenarios from the most common, such as walking along a lakeside, to out-of-the-ordinary experiences^1^. As an example, it is now possible to meet or interact with one’s DG, defined as “virtual humans that highly resemble the real self but behave independently”^2^. Most of the research involving DGs is related to training because some training approaches suggest that learning outcomes can be improved when exposing trainees to someone they feel similar to^4^.

Training using VR technology is developing in several fields, such as public speaking, pro-environmental behaviors, and health behaviors^3,10–15^. This development can be explained by the numerous advantages offered by this technology, such as the ease of access to training partners or the fact that training can be adapted over time by, for instance, progressively increasing the difficulty of the training depending on trainee progress^1^. Another advantage of VR-based training is that it allows trainees to be confronted with unexpected situations or situations that are impossible to replicate in real life, such that trainers can be designed to resemble the trainees.

#### Research involving doppelgangers

Research involving DGs is still scarce. Nonetheless, findings, mainly in a training setting, showed promising results^2–4,12^. An effective training program is composed of four phases, namely information, demonstration, practice, and feedback^16,17^. During the first phase, trainees receive information related to the desired behaviors to be learnt in a classroom format, in a written format, or both. During the second phase, trainees are shown the desired behavior by a role model. Traditionally, the demonstration phase relies on the use of videos in which trainees can watch examples of desired and undesired behaviors^16^. The two last phases consist in practicing the desired behavior and receiving feedback related to the practice phase for trainees to adjust their behavior in the desired way. These phases apply to all types of training ranging from learning a new sport to delivering a charismatic public speech. DGs, and more generally virtual humans, might be used in all or some of these phases. Virtual humans are multi-faceted trainers that might serve as: (a) instructors explaining what to do or not to do, (b) demonstrators showing what to do or not to do, and/or (c) partners with whom to train.

Past research mainly focused on the effect of DGs as role models in the demonstration phase, hence showing the benefit that using DGs might bring in a training setting. As an example, in the field of health behavior, Fox and Bailenson^3^ assessed the extent to which (not) observing one’s DG performing a physical effort and then losing or gaining weight (reinforcement condition vs. no change) can motivate individuals to engage subsequently in more physical exercises. Results showed a positive effect of the reinforcement condition (Study 1) and that observing one’s DG leads to a greater effect compared to observing a UA (Studies 2 and 3). In the field of sport, Bailenson et al.^12^ investigated the role of media (VR vs. video) in learning Tai Chi moves. Their main findings revealed greater outcomes in VR, that is, when participants could watch themselves performing the physical act between the learning sessions, than in the video setting. Studies 1 and 2 revealed an effect of media on subjective and objective learning, respectively.

#### The role of gender in research involving doppelgangers

Overall, empirical evidence showed that the use of a DG is effective and specifically in a training setting. Nonetheless, two studies in the field of public speaking^2,4^ revealed some discrepancies between female and male trainees. First, Aymerich-Franch and Bailenson^2^ investigated the extent to which DGs as role models (vs. visualization exercise) can reduce speaker stress before delivering a public speech. In the DG condition, participants were immersed in a virtual classroom and they first listened to a voice-over guiding a relaxation exercise (e.g., “Take a deep, comfortable breath and hold it”). Second, in VR they watched their DG delivering a speech while listening to the voice-over describing how successful they were during the speech (e.g., “Be aware of how well your speech is evolving”).

In the visualization condition, participants received the instructions to close their eyes and to listen to the same voice-over. The only difference with the DG condition is that they were asked to imagine themselves delivering the speech instead of watching their DG in VR. Next, participants completed a questionnaire and delivered their speech. Findings revealed that in the DG condition male participants reported lower levels of anxiety and higher communicative competence than female participants. Opposite results were found in the visualization condition such that female participants reported lower levels of anxiety and higher communicative competence than male participants.

Second, Kleinlogel et al.^4^ investigated the effectiveness of using DGs as role models in interpersonal skills training. Participants delivered two video-recorded speeches in front of a virtual audience. Before delivering the second speech, participants watched a role model, either their DG or a UA of the same gender, delivering a charismatic speech in front of the same audience. After the study, an independent coder (i.e., blind to the research question) evaluated participant performance. Results revealed that the DG-based training was the most beneficial for male trainees low in self-efficacy.

Kleinlogel et al.^4^ suggested that these gender effects might be explained in part by the public speaking context of these studies, which is a context that is traditionally associated with male prototypes^18^. It is plausible that stereotype threats are at work^19,20^, implying that there may be negative consequences in female attitudes and behaviors (e.g., lower performance). Another potential explanation is that women might be more disturbed by meeting their DG given that women tend to be more concerned by their appearance^6–9^. For instance, Muth and Cash^8^ investigated body-image attitudes among college students using questionnaires. Their main findings revealed that women reported more negative body-image evaluations (i.e., evaluations related to their overall body appearance), stronger body-image investments (i.e., importance and attention given to their appearance and engagement in appearance-managing behaviors), and more frequent body-image dysphoria (i.e., frequency of occurrence of negative body-image emotions in a series of 48 situations) than men. Results of a meta-analysis^7^ showed that these negative body-image emotions and attitudes can be explained in part by female exposure to mass media depicting thin-ideal bodies. Hence, by taking a stronger critical look at their appearance women would react to and perceive their DG negatively, which might negatively impact their attitudes and behaviors.

### The present research

Applied to our research, we investigate individuals’ emotional reactions (i.e., positive and negative emotions) toward and perceptions (i.e., competence and warmth) of a virtual human, which is either a DG or UA. We also assess whether gender matters by testing whether women and men react to and perceive the virtual person similarly depending on whether it resembles them or not.

#### Hypotheses

First, drawing on past research showing the effectiveness of DG in training-related research, we expect a main effect of the appearance of the virtual human (i.e., DG vs. UA). Furthermore, given the role played by engagement and motivation^21^ and attitudes toward the trainer^22^ on learning outcomes, it is plausible that the positive effect of DG-based training on learning outcomes stems from the positive impression that trainees have of their virtual self. The underlying mechanism might be that training with one’s virtual self is an unexpected situation which induces more positive feelings such as amusement or interest rather than negative feelings such as anxiety. A positive reaction and perception might foster a positive impression of the virtual trainer, which by extension might lead trainees to be more willing to imitate its behavior. Second, drawing on past research showing that DG-based training is less effective among women^2,4^, we expect an interaction effect between the virtual human appearance and participant gender.

Applied to our research and formally stated, we expect that individuals are more likely to feel positive emotions (Hypothesis 1a) and less likely to feel negative emotions (Hypothesis 1b) when meeting their DG as compared to a UA. We also expect that individuals are more likely to perceive their DG as competent (Hypothesis 1c) and warm (Hypothesis 1d) as compared to the UA. Furthermore, given that women tend to be more critical about their appearance^6–9^, we expect individuals’ gender to moderate these effects. We expect Hypothesis 1 to hold only for men and we expect to find the opposite results for women such that women are less likely to react positively and to perceive their DG positively as compared to the UA. Formally stated, we hypothesize that women are less likely to feel positive emotions (Hypothesis 2a), more likely to feel negative emotions (Hypothesis 2b), and less likely to perceive their DG as competent (Hypothesis 2c) and warm (Hypothesis 2d) than the UA.

#### Research design

To test our hypotheses, we used data from a research program composed of three studies conducted between 2016 and 2018 in our IVR laboratory. These studies were twofold. First, we conducted three experiments designed to assess the effectiveness of IVR-based training depending on whether the trainer was a DG or a UA of the same gender. We conducted three studies to investigate the extent to which the appearance of the trainer (DG or UA) influences the learning outcome in three different fields (i.e., training on pro-environmental behaviors, on empathic communication skills, and on public speaking skills) and whether different effects can be observed depending on the content of the training (see Methods section for more details about these studies).

The second aim was to investigate individuals’ emotional reactions to and perceptions of a virtual human while being immersed in VR. We collected the following data prior to collecting data related to the first aim of our research program. Prior to the training program, participants met the virtual trainer for two minutes, and then we measured participant reactions to and perceptions of that virtual human.

The present research includes the data from these three studies for two main reasons. First, merging data from three studies allowed for an increase in the sample size of our research to achieve sufficient statistical power. Accordingly, results of a sensitivity power analysis for multiple linear regression (fixed-model, R^2^ increase), using G*Power version 3.1^23^, suggest that even a small effect size (*f*^*2*^ = 0.046) can be detected with a sample size *N* = 310 (significance level α = 0.05, power 1 − β = 0.90, 3 tested predictors on a total of 5 predictors). Second, empirical evidence revealing a gender effect in the effectiveness of DG-based training stems from two studies in the field of public speaking. By merging three datasets from different fields, we aim to increase the generalizability of the results.

## Results

### Manipulation check

We performed a first set of analyses to assess whether participants in the DG condition identify with the virtual human to a greater extent than participants in the UA condition. Results showed that as expected participants who met their DG (*M* = 2.97, *SD* = 1.14) reported a higher level of identification than participants who met a UA (*M* = 2.62, *SD* = 1.14), *t*(308) = − 2.71, *p* = 0.003. We also checked whether female and male participants differ in their level of identification by performing a *t*-test per condition. Results revealed no gender difference in both the UA condition, *t*(157) = 1.25, *p* = 0.214, and the DG condition, *t*(149) = − 1.33, *p* = 0.184.

### Main analyses

Table 1 reports the mean, standard deviation, and correlation coefficients of the variables of interest. Table 2 reports the mean and standard deviation for each variable per condition and per participant gender. To test whether participants had similar reactions and perceptions toward either their DG or the UA, we performed a set of OLS hierarchical linear regression analyses^24^ using Stata version 16. First, we performed separate analyses using positive emotions, negative emotions, perceived competence, and perceived warmth as the dependent variables and, in each analysis, we entered the variables Conditions (0 = UA, 1 = DG) and participant Gender (0 = female, 1 = male). We controlled for the study design by including two dummy variables to take into account any difference between the three studies. Second, we replicated these four regression analyses by adding the interaction term Conditions x Gender to our models to test the hypothesized interaction effects. Table 3 reports the results of these analyses.

**Table 1:** Means, standard deviations, and correlations.

Variables	Mean	Std. Dev	1	2	3	4	5	6
1. Perceived warmth	5.57	1.74						
2. Perceived competence	6.20	1.32	.63***					
3. Positive emotions	5.41	1.79	.33***	.26***				
4. Negative emotions	4.02	1.75	− .24***	− .07	− .02			
5. Identification	2.79	1.15	.20***	.19***	.21***	.02		
6. Conditions	–	–	− .17***	− .12**	.08	.20***	.15***	
7. Gender	–	–	− .10*	− .09*	− .06	.03	.01	.02

*p < .05; ** p < .01; *** p < .001. Conditions coded 0 for UA (Unknown Avatar) condition and 1 for DG (Doppelganger) condition; Gender coded 0 for female participants and 1 for male participants.

**Table 2:** Mean and standard deviation of each of the variables per condition and per participant gender.

	Conditions				Gender			
	UA		DG		Female		Male	
	*M*	*SD*	*M*	*SD*	*M*	*SD*	*M*	*SD*
Perceived warmth	5.86	1.91	5.27	1.48	5.76	1.97	5.41	1.50
Perceived competence	6.35	1.37	6.04	1.25	6.33	1.41	6.09	1.24
Positive emotions	5.27	1.68	5.55	1.90	5.51	1.78	5.32	1.80
Negative emotions	3.67	1.79	4.38	1.64	3.95	1.76	4.07	1.74
Identification	2.62	1.14	2.97	1.14	2.79	1.21	2.80	1.11

**Table 3:** Results of the regression analyses related to our four dependent variables.

	Positive emotions		Negative emotions		Perceived competence		Perceived warmth	
	(1)	(2)	(1)	(2)	(1)	(2)	(1)	(2)
Dummy 1	− 0.05	− 0.05	− 0.08	− 0.08	0.30***	0.30***	0.25***	0.25***
	(− 0.65)	(− 0.63)	(− 1.12)	(− 1.11)	(4.49)	(4.48)	(3.68)	(3.75)
Dummy 2	0.21**	0.21**	− 0.24***	− 0.24***	0.23***	0.23***	0.32***	0.32***
	(2.91)	(2.94)	(− 3.31)	(− 3.31)	(3.30)	(3.33)	(4.63)	(4.68)
Conditions	0.08	− 0.08	0.20***	0.18*	− 0.11*	− 0.27***	− 0.16**	− 0.43***
	(1.48)	(− 0.99)	(3.60)	(2.16)	(− 1.99)	(− 3.24)	(− 2.98)	(− 5.00)
Gender	− 0.06	− 0.21**	0.02	0.01	− 0.06	− 0.20**	− 0.07	− 0.31***
	(− 1.14)	(− 2.89)	(0.42)	(0.11)	(− 1.13)	(− 2.64)	(− 1.35)	(− 3.80)
Conditions × Gender		0.27**		0.03		0.26**		0.43***
		(2.71)		(0.27)		(2.69)		(4.55)
*N*	310	310	310	310	310	310	310	310
*F*	6.178***	6.723***	7.671***	6.157***	6.731***	6.893***	9.102***	12.24***
*R*^*2*^	0.067	0.089	0.080	0.081	0.081	0.102	0.107	0.165
∆*R*^*2*^		0.022**		0.001		0.021**		0.058***

*p < .05; **p < .01; ***p ≤ .001. Conditions coded 0 for UA (Unknown Avatar) condition and 1 for DG (Doppelganger) condition; Gender coded 0 for female participants and 1 for male participants; Dummy 1 and Dummy 2 are included to control for the design of the three studies composing the research; Robust *t*-statistics in parentheses; Estimates are standardized (OLS estimation).

#### Emotional reactions to the virtual trainer

**Figure 1:**
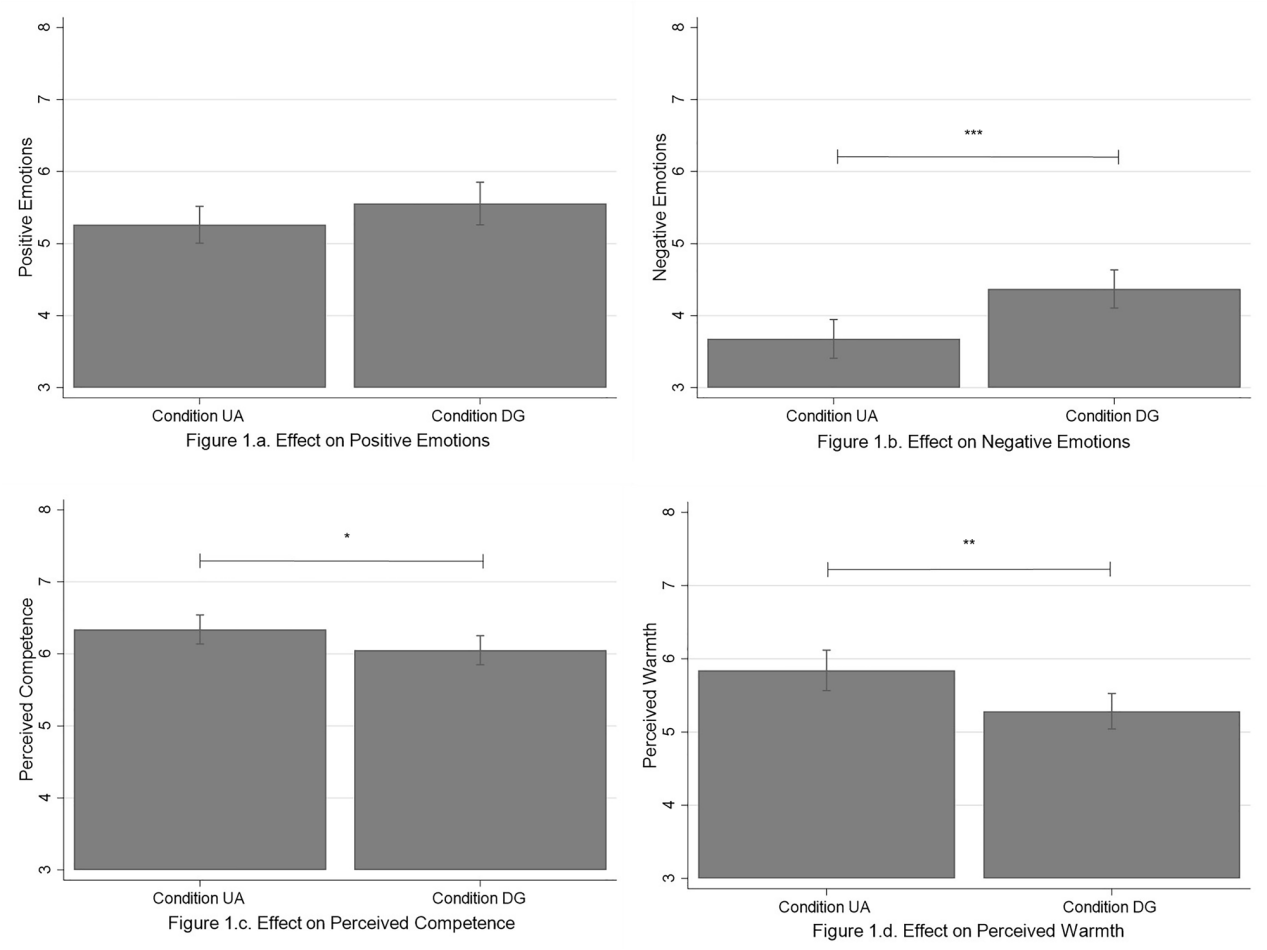
Effect of Conditions on the dependent variables. *Note*: Condition UA = Unknown avatar; Condition DG = Doppelganger. Bar plots reporting the predictive margins of conditions with the 95% CIs (confidence intervals).

**Figure 2:**
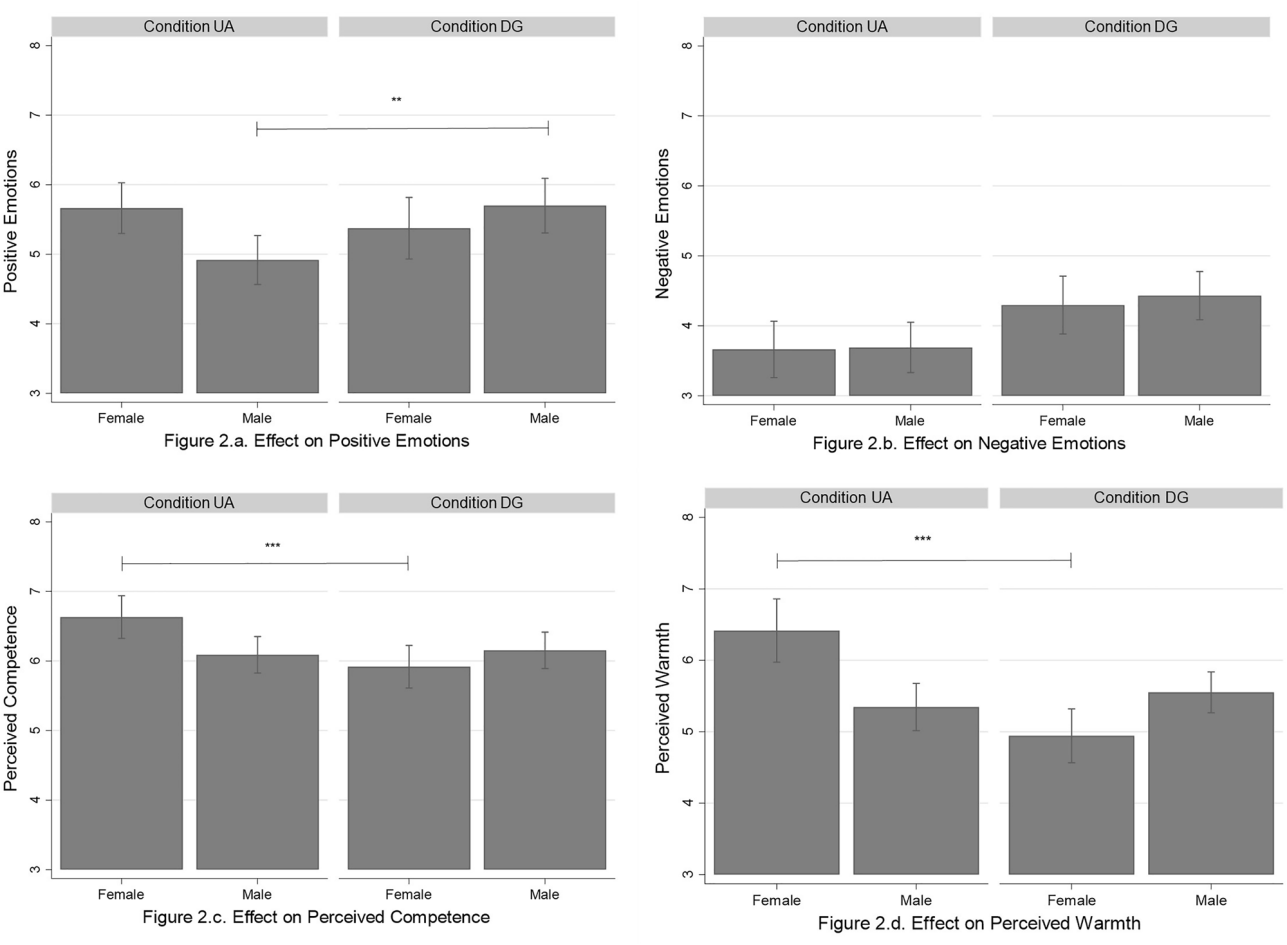
Interaction effect of Conditions × Gender on the dependent variables. *Note*: *p < .05; **p < .01; ***p ≤ .001. Condition UA = Unknown avatar; Condition DG = Doppelganger. Bar plots reporting the predictive margins of conditions × gender with the 95% CIs (confidence intervals).

Figure 1a–d are graphical representations of the effect of Conditions on our dependent variables. Figure 2a–d are graphical representations of the interaction effect of Conditions x Gender on our dependent variables. First, results did not show any significant main effect with regards to positive emotions (see Fig. 1a). However, we found a significant and positive main effect of Conditions on negative emotions (*β* = 0.20, *p* < 0.001), revealing that participants who met their DG felt more negative emotions than those who met the UA (see Table 3 and Fig. 1b). These results did not support Hypotheses 1a and 1b. Finally, results did not reveal any significant main effect of Gender.

The second set of analyses revealed a significant Conditions x Gender interaction on positive emotions (*β* = 0.27, *p* = 0.007; see Table 3). We performed simple slopes analyses^24^ to probe this interaction effect and test Hypothesis 2a. Follow-up analyses showed that male participants in the DG condition felt more positive emotions than male participants in the UA condition (*β* = 0.78, *p* = 0.004). Results also showed that female participants felt a similar level of positive emotions in both conditions (*β* = -0.29, *p* = 0.321), not supporting Hypothesis 2a (see Fig. 2a). We did not find any significant interaction with regards to negative emotions, thus Hypothesis 2b was not supported (see Fig. 2b).

#### Perceptions of the virtual trainer

To assess whether participants perceived the virtual trainer similarly in terms of competence and warmth depending on Conditions, we replicated the above analyses using the variables of perceived competence and perceived warmth as dependent variables, respectively. Results revealed a significant and negative main effect of Conditions on perceived competence (*β* = − 0.11, *p* = 0.047) and perceived warmth (*β* = − 0.16, *p* = 0.003), not supporting Hypotheses 1c and 1d (see Table 3). These findings showed that participants in the DG condition perceived the virtual human as less competent and less warm than participants in the UA condition (see Fig. 1c and d). Results did not reveal any significant main effect of Gender.

Finally, we found a significant Conditions x Gender interaction on both perceived competence (*β* = 0.26, *p* = 0.008) and perceived warmth (*β* = 0.43, *p* < 0.001; see Table 3). Simple slopes analyses showed that female participants in the DG condition perceived the virtual human as less competent (*β* = − 0.71, *p* = 0.001) and less warm (*β* = − 1.47, *p* < 0.001) than female participants in the UA condition (see Fig. 2c and d), supporting Hypotheses 2c and 2d. Follow-up analyses also showed that male participants reported similar levels of perceived competence (*β* = 0.06, *p* = 0.730) and warmth (*β* = 0.20, *p* = 0.358) in both conditions.

## Discussion

The present work reveals that participant reactions toward and perceptions of a virtual human were influenced by its appearance (DG vs. UA). We mainly found that participants felt more negative emotions and perceived it as less competent and warm when it resembled them. Further analyses revealed that the effects related to participant perceptions of the virtual human were moderated by participant gender. Women perceived their DG as less competent and warm than the UA, whereas men had a similar perception of the DG or the UA. Finally, results showed that men felt more positive emotions when meeting their DG (vs. UA). Overall, our findings suggest that women tend to be highly critical of their DG, whereas men tend to enjoy and be amused by this experience.

First, from a theoretical point of view, our work contributes to a better understanding of the emotional reactions and perceptions that individuals have when meeting a virtual human. We found that these reactions and perceptions were affected by the virtual human’s appearance, such that meeting one’s DG (vs. UA) induces negative reactions and perceptions. Importantly, findings revealed that part of these effects was driven by participant gender such that negative perceptions of the DG were observed only among women. These findings echo past research showing that women tend to have a negative image of their appearance^6–9^. Despite the fact that women did not feel more positive or negative emotions when meeting their DG, they perceived it as lacking competence and warmth. These findings might provide a first potential explanation as to why past research^2,4^ found that public speaking training involving a DG was mainly beneficial to men.

Second, our findings have important practical implications in terms of IVR-based training and research design. In a training setting, our work suggests that female trainees might not benefit, as much as male trainees, from participating in DG-based training due to the negative perceptions caused by meeting one’s virtual self. Trainees should be able to feel comfortable during a training session such that they can learn in a safe and favorable context. By perceiving the virtual trainer negatively, it is plausible to argue that female trainees are less likely to engage in the training (e.g., they may be less involved, more reluctant to participate, more focused on the appearance of the DG instead of focusing on the content of the training program). In a more general research setting, participants should be able to feel comfortable during a study, such that they can behave in a natural way. Hence, our research should sensitize researchers that the effects induced by the use of DGs might depend on the characteristics of the participants, and that in some cases (i.e., female participants) the use of DGs might affect their IVR-based experience.

Future research is needed to give clear recommendations for study design involving the use of DGs. As a next step, it seems crucial to assess whether these negative perceptions might influence research outcomes and whether these perceptions can depend on the research topic. Related to this point, it would be interesting to assess whether the use of a DG might affect outcomes in fields other than training and learning, such as whether a DG might affect women’s attitudes and behaviors during a metaverse meeting.

In the specific setting of IVR-based training research, we call on future research to replicate the present work, as well as to further investigate this phenomenon by assessing the extent to which female trainees’ negative perceptions of their DG might affect their learning outcome. It is plausible to argue that the observed negative perceptions would lower female learning outcome, which might explain why past research found that public speaking training using a DG was only beneficial to male trainees. A more complex mechanism could be at play when investigating trainee perceptions and learning outcome, such that the negative perceptions of female trainees might first influence their attitudes towards the training, which then influence their learning process, and hence influence the learning outcome. As an example, future research might assess the extent to which trainee perceptions of the trainer and the learning outcome are related to trainee involvement and motivation to participate in the training program (measured before and after exposure to the virtual trainer). Collecting qualitative data related to this phenomenon would provide additional information related to the observed gender effects found in the present work and in previous research. It is noteworthy that investing in developing DGs is costly, hence it is crucial to understand the extent to which the use of DGs might be beneficial to trainees or whether this benefit might only matter for half of the population (i.e., men).

As a main limitation, it is important to note that we used tools to create virtual human avatars that are quite realistic. However these tools had some drawbacks. For instance, we could not model participant haircuts, which might have influenced participant reactions and perceptions, and more specifically those of women. Nonetheless, to date new high-performance tools are available to create avatars, which might lead to even more realistic and better quality results, including haircut renditions (e.g., Crazytalk, Headshot).

## Method

### Participants

The sample consisted of 310 students from three different studies (*M*_*age*_ = 21.48, *SD* = 2.97, 45% women). Students were all enrolled in a program at a Swiss university and were mainly undergraduate students (77%). Table 4 reports the description of the sample for each study. Participants received from CHF 40 (about USD 44) to CHF 80 (about USD 88) as compensation for their participation, depending on the study duration. The studies were performed in line with the principles of the Declaration of Helsinki. Approval for each study was granted by the Commission for Ethics in Research (CER-HEC) at the Swiss university in which we conducted the studies. Prior to their participation, participants obtained information about the study (e.g., duration, compensation, data confidentiality, voluntary participation, and possibility to withdraw at any time without needing to provide a reason and without cost) and signed an informed consent form with the corresponding information.

**Table 4:** Description of each study sample.

Field of Study	*N*	*M*_age_ (*SD*)	% women (%)	% undergraduate students (%)
Pro-environmental behavior	107	21.73 (3.91)	45	82
Empathic communication	127	21.41 (2.25)	51	70
Public speaking	76	21.24 (2.49)	37	80
All studies	310	21.48 (2.97)	45	77

Given that we investigated the gender effect on our dependent variables in our study, before merging our three datasets we performed a Pearson Chi-square test to assess whether participant gender was equally distributed across the three studies. Results from the Chi-square test showed that gender proportions did not differ significantly between the three studies, χ^2^ (2) = 3.97, *p* = .138.

### Overview of the three studies

The first aim of the three studies was to investigate the effectiveness of IVR-based training depending on whether the trainer was a DG or a UA. To assess the training effectiveness, we measured the learning outcome by collecting data on participant behavior before and after the training session using online questionnaires or through video ratings. The learning outcome was specific to each study. In the first study, we investigated participant pro-environmental behaviors^25^. In IVR, participants received energy-saving instructions from a virtual human (DG or UA). For each instruction, the virtual human showed appropriate gestures. For instance, she/he pointed at the fridge when explaining how to save energy related to fridge management (see^25^ for pictures of the virtual environment). To capture participant learning outcome, participants filled in online questionnaires related to their pro-environmental norms, attitudes, and behaviors.

In the second study, we investigated participant communication skills. Before and after the training session, we asked participants to place themselves in the shoes of a human resources employee, and to deliver bad news to virtual employees in a desktop setting while being videotaped. Each time, they delivered bad news to three different employees (in random order) who had different emotional reactions (anger, sadness, and denial). In each situation, participants had to react and respond to the employee by adapting their behavior. In total, there were three interactions between each employee and the participant. For instance, in the anger reaction scenario, the employee reacted to the bad news by using an aggressive tone and said (1) “Um… I am not sure that I understand what you just said! Are you telling me that I am fired?! You are thanking me for all my good work by firing me?!”, (2) “It was so hard for me to find this job… and I have been working hard ever since I started working in this company to make sure that you will keep me. I have made personal sacrifices by staying late in the evening and by working on some weekends. Can you explain why you are firing me because I really do not get it!”, and finally (3) “I still do not get it.” Between the two tasks, participants took part in a training session in IVR aiming at improving their empathic communication skills when delivering bad news. Specifically, they practiced these skills with a virtual human (DG or UA). To capture participant learning outcome, we asked an independent coder to rate the extent to which participants communicated the bad news empathetically (verbal and nonverbal behaviors) by watching their videotapes before and after the training session.

In the third study, we investigated participant public speaking skills^4^. Specifically, this study focused on nonverbal behavior. First, participants were asked to deliver a speech in front of an audience in IVR while being videotaped. The audience was composed of more than 100 virtual humans seated in a large conference room and carefully listening to the speech (see^4^ for pictures of the room). Participants were on a stage facing the audience to deliver their speech. After delivering their speech, participants took part in a training session consisting in a demonstration of a perfect speech: They watched a virtual human (DG or UA) giving an impressive charismatic speech on the same topic in front of the same audience. Next, participants delivered their speech a second time. We captured the learning outcome by asking an independent coder to watch the videotapes and to rate the appropriateness of participant gestures, body openness, and body language expressiveness.

In each of the three studies, prior to the training, participants met the virtual human for two minutes. At this stage of the studies, participants knew that they were participating in a research project either on pro-environmental behaviors, on empathic communication, or on public speaking skills because prior to this meeting they had to do something or to fill in questionnaires composed of measures related to the learning outcome. In the three studies, participants also knew that they were going to meet the virtual human that was then part of the training phase. They were informed that the virtual human would give them energy saving instructions, would be the employee receiving the bad news, or would give a public speech that they would be attending. The only difference in the instructions between the three studies is that we informed participants from the third study that the virtual human they were to meet was either an avatar or an avatar that resembled the participant depending on the assigned condition. We did not provide this information to the participants from the two other studies. We did so in the third study to render the virtual human appearance (DG vs. UA) manipulation more salient.

### Materials and procedure

Each study had a similar setting and participants came at least twice to the laboratory. The main goal of Session 1 was to take photos of each participant’s face (front and side view). Prior to Session 2, we randomly assigned participants to one of the two experimental conditions of the studies such that the virtual human involved in the training session was either their doppelganger (*N* = 159, DG condition) or an unknown avatar (*N* = 151; UA condition). In Session 2, prior to the training phase in IVR, participants were immersed in a virtual room and met the virtual human involved in the training session (DG or UA). During this meeting, the main task the participants were asked to complete was to look at the virtual human for two minutes. Next, the experimenter read out loud a series of adjectives designed to capture emotional reactions of the participants and their perceptions of the virtual human while they were still facing it in IVR. Adjectives were presented in a randomized order in two studies. In the third study, we audio-recorded the adjectives for convenience purposes, implying that all study participants were provided with the adjectives in the same order. For each adjective, participants said how they felt or perceived the virtual human. Next, they took part in the training session designed to increase pro-environmental behaviors^25^, to develop skills to communicate bad news with empathy, or to develop public speaking skills^4^.

**Figure 3:**
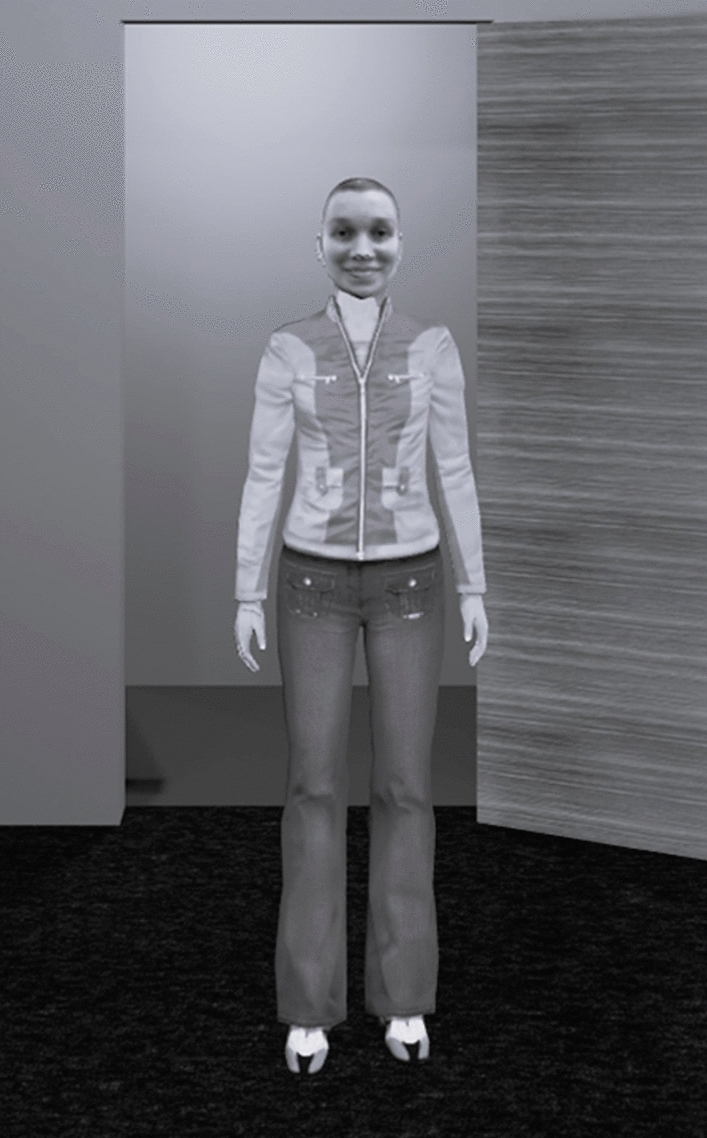
Female unknown avatar that female participants in the UA condition met. *Note*: UA = Unknown avatar.

The virtual environment related to the meeting was developed using Vizard from WorldViz. The virtual humans for the DG and UA conditions were developed using 3ds Max toolkit. The doppelgangers were created using participants’ photos collected during Session 1. The unknown avatars were created using photos of a male and a female lab member (unfamiliar to the participants). This enabled us to ensure that the virtual humans in both conditions were of similar quality and to ensure that participant gender matched the virtual human's gender. To illustrate, Fig. 3 shows the unknown avatar that female participants in the UA condition met.

### Measures

#### Emotional reactions to the virtual trainer

Participants reported how they felt with respect to the virtual human by using a 10-point scale ranging from 1 (*not at all*) to 10 (*absolutely*). Nine adjectives were used (i.e., enthusiastic, delighted, amused, interested, impressed, confused, skeptical, anxious, and disturbed). We averaged the five adjectives related to positive emotions (*M* = 5.41, *SD* = 1.79, α = 0.83) and averaged the four adjectives related to the negative emotions (*M* = 4.01, *SD* = 1.75, α = 0.76).

#### Perceptions of the virtual trainer

We measured the extent to which participants perceived the virtual human as competent and warm^26,27^ on a scale ranging from 1 (*not at all*) to 10 (*absolutely*). We measured perceived competence using the three adjectives (competent, confident, and intelligent) and perceived warmth using three adjectives (friendly, warm, and inspired confidence). We averaged the three items for competence (*M* = 6.20, *SD* = 1.32, α = 0.73) and for warmth (*M* = 5.57, *SD* = 1.74, α = 0.82).

#### Identification with the avatar

We assessed the extent to which participants identified with the virtual human on a 5-point Likert-type scale, ranging from 1 (*strongly disagree*) to 5 (*strongly agree*). We used the item “I identified with the virtual person” (*M* = 2.79, *SD* = 1.15).

## Data Availability

The datasets generated during and/or analysed during the current study are available from the corresponding author on reasonable request.
